# Artesunate: could be an alternative drug to chloroquine in COVID-19 treatment?

**DOI:** 10.1186/s13020-020-00336-8

**Published:** 2020-05-28

**Authors:** Tuğçenur Uzun, Orcun Toptas

**Affiliations:** 1 Department of Oral and Maxillofacial Surgery, Trabzon Oral and Dental Health Hospital, DDS, Trabzon, Turkey; 2 Department of Oral and Maxillofacial Surgery, Faculty of Dentistry, Abant Izzet Baysal University, Bolu, Turkey

**Keywords:** SARS-CoV-2, Choroquine, Artesunate

## Abstract

SARS (Severe Acute Respiratory Syndrome Coronavirus)-CV-2 (2019-nCov), which showed up in China in December 2019 and spread all over the world, has becomed a serious health problem. An effective, safe and proven treatment has not yet been found. Chloroquine has been recommended by some authors to be used for the treatment of patients infected with this virus however chloroquine may have side effects and drug resistance problems. Artesunate is a semisynthetic derivative of artemisinin, an antimalarial drug. Artesunate was thought to be an effective treatment for covid-19 because of its anti-inflammatory activity, NF-κB (nuclear Factor kappa B)-coronavirus effect and chloroquine-like endocytosis inhibition mechanism.

## Background

SARS-CoV-2 (2019-nCov), which showed up in China in December 2019 and spread all over the world, has become a serious health problem. Concomitant diseases and older age increase the risk of mortality. An effective, safe and proven treatment has not yet been found. Chloroquine/hydroxychloroquine used for the treatment of malaria inhibits the replication of many DNA and RNA viruses, including human coronaviruses [1]. Chloroquine has been shown to inhibit SARS-CoV-2 in vitro and has been recommended by some authors to be used for the treatment of patients infected with this virus [2, 3]. Thanks to the weak base properties, chloroquine and hydroxychloroquine increase acidic the pH of intracellular organelles such as endosome/lysosome and prevent enveloped viruses such as coronavirus from penetrating into the cell. In long-term use, chloroquine may have side effects such as retinopathy and cardiomyopathy [4–6]. This has led to the search for a safer drug with antiviral and immunomodulatory properties that can be used for the treatment of covid-19. Artesunate is a semisynthetic derivative of artemisinin, an antimalarial drug, which is obtained from the plant Artemisia annua L. Artemisia annua L. is a plant that has been used in traditional Chinese medicine for centuries. Artemisinin has also been defined by the World Health Organization as “the best hope for malaria treatment”. In a study published in 2004, Hoppe et al. mentioned the decreasing clinical benefits and toxic properties of quinolone antimalarials chloroquine and mefloquine due to the growing parasite resistance, and have emphasized that artemisinin is a highly potent antimalarial drug and can overcome the resistance problems experienced with the quinoline drugs. They also demonstrated in this study that artemisinin inhibited endocytosis more strongly than chloroquine, and unlike chloroquine, did not cause inhibition of transport vesicle-vacuole fusion [7].

Artesunate may inhibit NF-kB (Nuclear Factor kappa B) downregulation and viral protein synthesis, disrupting the early phase of viral replication [8, 9]. Kaptein et al. showed in their study published in 2006 that artesunate inhibited the replication of cytomegalovirus in vivo and in vitro [10]. Artesunate has the highest antiviral activity against HCMV (Human Cytomegalovirus) among the derivatives of artemisinin [11]. Artemisinin/artesunate has been shown to inhibit the reproduction of hepatitis B virus in vitro [12]. Artemisinin also inhibits the replication of hepatitis C replicon, which, just like SARS-CoV-2, is a single-stranded RNA virus [13]. Dai et al. found in their study in 2015 that artesunate inhibits hepatitis C replication in vitro better than ribavirin but worse than interferon-2b(IFN), while the combination of IFN and artesunate showed synergistic effects [14]. Sharma et al. demonstrated artesunate to inhibit the replication of JC polyomavirus (involved in the pathogenesis of progressive multifocal leukoencephalopathy) in vitro [15].

Coronavirus primarily involves the upper respiratory tract and causes an increase in the host immune response [16]. The inflammatory response increases with the activation of NF-κB in the host infected with coronavirus, and the production of proinflammatory cytokines and chemokines significantly affects the course of the disease [17, 18]. The inflammatory response in SARS-CoV-2 infection has not been elucidated completely, and uncontrolled release of inflammatory cytokines is thought to be responsible for the pathogenesis of the disease. The SARS-CoV-2 infection causes a fatal inflammatory response and acute lung injury [19]. Being an important regulator of host immune responses against invading pathogens, NFκB has been shown to be affected in both MERS-CoV and SARS-CoV infections [17, 20]. Christman et al. reported that NF-κB has a key role in the pathogenesis of many lung diseases [21]. Artesunate demonstrates its anti-inflammatory activities over NF-kB. Artesunate is thought to inhibit IL-1β (interleukin-1beta), IL-6 (interleukin 6) and IL-8 (interleukin 8) production through inhibition of the NF-κB signal pathway [22].

Increased secretion of IL-1β, IFN-γ (Interferon gamma), IP-10 (induced protein 1), MCP-1 (Monocyte chemoattractant protein-1), IL-4 (interleukin 4), and IL-10 (interleukin 10) secretion is seen in patients infected with SARS-Cov-2 [23]. One ex vivo experiment by Chu et al. demonstrated SARS-CoV-2 to cause an upregulation of IL6, MCP1, CXCL1 [chemokine (C-X-C motif) ligand 1], CXCL5 [chemokine (C-X-C motif) ligand 5] and CXLC10 [chemokine (C-X-C motif) ligand 10, (IP10)] [24]. Research suggests that elevated serum IL-6 levels may constitute a biomarker for severe disease progression [25]. IL-6 has a crucial role in cytokine release syndrome (CRS), which occurs during SARS-CoV-2 infection, suggesting that controlling IL-6 can affect the course of the disease positively [26]. Among patients infected with SARS-CoV-2, those with severe disease progression have been shown to have significantly higher levels of IL-2 (interleukin 2), IL-7 (interleukin 7), IL-10, G-CSF (Granulocyte-colony stimulating factor), IP-10, MCP1, MIP1a (Macrophage inflammatory protein 1-alpha) and TNF-α (tumor necrosis factor-alpha) compared to those with a mild course [23]. Li et al. reported in their study published in 2020 reviewing coronavirus infection and immune response that viral infections triggered the host immune response; however, increased and uncontrolled immune response was responsible for the pathogenesis of the disease. In cases of Cov pneumonia, control of cytokine production and inflammatory response may exhibit a positive effect by reducing cell and fluid collection [27].

Artemisinin and its derivatives have anti-inflammatory and immune regulatory effects [28]. Artesunate has been reported to be effective in systemic lupus erythematosus, rheumatoid arthritis and allergic contact dermatitis [22, 29, 30]. It has been demonstrated that artemisinin and its derivatives suppress the production of IL-2, inhibit nitric oxide synthase and NF-κB activation, thereby providing the treatment of rheumatoid arthritis [31, 32]. It has been demonstrated that artesunate can regulate the effects of regulator T cells via NF-κB/p65 and Smad2/3-dependent TGF-β (Transforming growth factor beta) signaling [33]. Jiang et al. reported that artesunate showed anti-inflammatory activity by causing a decrease in TNF-α and IL-6 levels [34]. Mo et al. found artesunate to significantly reduce th expression of MCP-1 and TNF-α in serum [35].

Middle East Respiratory Syndrome Coronavirus (MERS-CoV) was first isolated in 2012 and is a virus believed to have evolved from bat coronaviruses such as SARS-CoV and SARS-CoV-2 [36, 37]. Since NF-κB is an important regulator of host immune responses against invading pathogens, many viral proteins have been shown to affect NF-κB, including MERS-CoV proteins. Canton et al. reported that MERS-CoV 4b protein was required for the inhibition of NF-κB activation in MERS-CoV infection [38, 39]. While NF-κB is seen mostly outside the nucleus during infection, ORF4b has been found to be localized in the nucleus. Moreover, in the absence of functional ORF4b protein, NF-κB could pass into the nucleus and express pro-inflammatory cytokines such as TNF-α and IL-8. Furthermore, it has been demonstrated that in the cytoplasm, MERS-CoV 4b protein interacts with karyopherin-α4, an importin α2 family member, in a nuclear localization signal (NLS)-dependent manner, resulting in its inability to bind to a subunit (p65) of NF-κB. Considering the roles of NF-κB in not only innate but also adaptive immune responses, it is still likely that other MERS-CoV proteins may target NF-κB to alter the host immune response. Indeed, in another study, MERS-CoV-derived ORF4a and ORF8b proteins have been shown to antagonize NF-κB [20]. Therefore, there is a possibility that SARS-CoV-2 may increase its activity through NF-κB inhibition during the infection, as in MERS-CoV from the same virus family.

## Conclusion

Artesunate was thought to be an effective treatment for covid-19 because of its the above-mentioned anti-inflammatory activity, NF-κB-coronavirus effect and chloroquine-like endocytosis inhibition mechanism.

## Data Availability

Not applicable.

## Abbreviations

SARS: Severe acute respiratory syndrome coronavirus
NF-κB: Nuclear factor kappa B
TNF-α: Tumor necrosis factor Alpha
IL-6: Interleukin 6
IL-1β: Interleukin 1β
IFN-γ: Interferon gamma
IP-10: Induced protein 1
MCP-1: Monocyte chemoattractant protein-1
IL-4: Interleukin 4
IL-10: Interleukin 10
IL-1beta: Interleukin-1beta
IL-8: Interleukin 8
NLS: Nuclear localization signal
TGF-β: Transforming growth factor
IFN: Interferon-2b
HCMV: Human cytomegalovirus
JC: John Cunningham
CXCL1: Chemokine (C-X-C motif) ligand 1
CXCL5: Chemokine (C-X-C motif) ligand 5
CXLC10: Chemokine (C-X-C motif) ligand 10, (IP10)
IL2: Interleukin 2
IL7: Interleukin 7
G-CSF: Granulocyte-colony stimulating factor
MIP1a: Macrophage inflammatory protein 1-alpha

## References

[1] Devaux CA, Rolain JM, Colson P, Raoult D (2020). New insights on the antiviral effects of chloroquine against coronavirus: what to expect for COVID-19?. Int J Antimicrob Agents.

[2] Wang M, Cao R, Zhang L, Yang X, Liu J, Xu M (2020). Remdesivir and chloroquine effectively inhibit the recently emerged novel coronavirus (2019-nCoV) in vitro. Cell Res.

[3] Colson P, Rolain JM, Raoult D (2020). Chloroquine for the 2019 novel coronavirus SARS-CoV-2. Int J Antimicrob Agents.

[4] Bernstein HN (1991). Ocular safety of hydroxychloroquine. Annals Ophthalmology.

[5] Ratliff NB, Estes ML, Myles JL, Shirey EK, McMahon JT (1987). Diagnosis of chloroquine cardiomyopathy by endomyocardial biopsy. N Eng J Med.

[6] Iglesias Cubero G, Rodriguez Reguero JJ, Rojo Ortega JM (1993). Restrictive cardiomyopathy caused by chloroquine. Br Heart J.

[7] Hoppe HC, van Schalkwyk DA, Wiehart UIM, Meredith SA, Egan J, Weber BW (2004). Antimalarial quinolines and artemisinin inhibit endocytosis in *Plasmodium falciparum*. Antimicrob Agents Chemother.

[8] Efferth T, Romero MR, Wolf DG, Stamminger T, Marin JJ, Marschall M (2008). The antiviral activities of artemisinin and artesunate. Clin Infect Dis.

[9] Drouot E, Piret J, Boivin G (2016). Artesunate demonstrates in vitro synergism with several antiviral agents against human cytomegalovirus. Antiviral Therapy.

[10] Kaptein SJ, Efferth T, Leis M, Rechter S, Auerochs S, Kalmer M (2006). The anti-malaria drug artesunate inhibits replication of cytomegalovirus in vitro and in vivo. Antiviral Res.

[11] D’Alessandro S, Scaccabarozzi D, Signorini L, Perego F, Ilboudo DP, Ferrante P (2020). The use of antimalarial drugs against viral infection. Microorganisms.

[12] Romero MR, Efferth T, Serrano MA, Castano B, Macias RI, Briz O (2005). Effect of artemisinin/artesunate as inhibitors of hepatitis B virus production in an “in vitro” replicative system. Antiviral Res.

[13] Obeid S, Alen J, Nguyen VH, Pham VC, Meuleman P, Pannecouque C (2013). Artemisinin analogues as potent inhibitors of in vitro hepatitis C virus replication. PLoS ONE.

[14] Dai R, Xiao X, Peng F, Li M, Gong G (2016). Artesunate, an anti-malarial drug, has a potential to inhibit HCV replication. Virus Genes.

[15] Sharma BN, Marschall M, Rinaldo CH (2014). Antiviral effects of artesunate on JC polyomavirus replication in COS-7 cells. Antimicrob Agents Chemother.

[16] Brian DA, Baric RS (2005). Coronavirus genome structure and replication. Curr Top Microbiol Immunol.

[17] DeDiego ML, Nieto-Torres JL, Regla-Nava JA, Jimenez-Guardeno JM, Fernandez-Delgado R, Fett C (2014). Inhibition of NF-kappaB-mediated inflammation in severe acute respiratory syndrome coronavirus-infected mice increases survival. J Virol.

[18] Kanzawa N, Nishigaki K, Hayashi T, Ishii Y, Furukawa S, Niiro A (2006). Augmentation of chemokine production by severe acute respiratory syndrome coronavirus 3a/X1 and 7a/X4 proteins through NF-kappaB activation. FEBS Lett.

[19] Fu Y, Cheng Y, Wu Y (2020). Understanding SARS-CoV-2-mediated inflammatory responses: from mechanisms to potential therapeutic tools. Virologica Sinica.

[20] Lee JY, Bae S, Myoung J (2019). Middle east respiratory syndrome coronavirus-encoded accessory proteins impair MDA5-and TBK1-mediated activation of NF-kappaB. J Microbiol Biotechnol.

[21] Christman JW, Sadikot RT, Blackwell TS (2000). The role of nuclear factor-kappa B in pulmonary diseases. Chest.

[22] Xu H, He Y, Yang X, Liang L, Zhan Z, Ye Y (2007). Anti-malarial agent artesunate inhibits TNF-alpha-induced production of proinflammatory cytokines via inhibition of NF-kappaB and PI3 kinase/Akt signal pathway in human rheumatoid arthritis fibroblast-like synoviocytes. Rheumatology.

[23] Huang C, Wang Y, Li X, Ren L, Zhao J, Hu Y (2020). Clinical features of patients infected with 2019 novel coronavirus in Wuhan, China. Lancet.

[24] Chu H, Chan JF, Wang Y, Yuen TT, Chai Y, Hou Y (2020). Comparative replication and immune activation profiles of SARS-CoV-2 and SARS-CoV in human lungs: an ex vivo study with implications for the pathogenesis of COVID-19. Clin Infect Dis.

[25] Herold T, Jurinovic V, Arnreich C, Hellmuth JC, von Bergwelt-Baildon M, Klein M (2020). Level of IL-6 predicts respiratory failure in hospitalized symptomatic COVID-19 patients. medRxiv.

[26] Zhang C, Wu Z, Li J-W, Zhao H, Wang G-Q (2020). The cytokine release syndrome (CRS) of severe COVID-19 and Interleukin-6 receptor (IL-6R) antagonist Tocilizumab may be the key to reduce the mortality. Int J Antimicrob Agents.

[27] Li G, Fan Y, Lai Y, Han T, Li Z, Zhou P (2020). Coronavirus infections and immune responses. J Med Virol.

[28] Efferth T (2006). Molecular pharmacology and pharmacogenomics of artemisinin and its derivatives in cancer cells. Curr Drug Targets.

[29] Adjuik M, Babiker A, Garner P, Olliaro P, Taylor W, White N (2004). Artesunate combinations for treatment of malaria: meta-analysis. Lancet.

[30] Chen H, Maibach HI (1994). Topical application of artesunate on guinea pig allergic contact dermatitis. Contact Dermatitis.

[31] Aldieri E, Atragene D, Bergandi L, Riganti C, Costamagna C, Bosia A (2003). Artemisinin inhibits inducible nitric oxide synthase and nuclear factor NF-kB activation. FEBS Lett.

[32] He Y, Fan J, Lin H, Yang X, Ye Y, Liang L (2011). The anti-malaria agent artesunate inhibits expression of vascular endothelial growth factor and hypoxia-inducible factor-1alpha in human rheumatoid arthritis fibroblast-like synoviocyte. Rheumatol Int.

[33] Li T, Chen H, Yang Z, Liu XG, Zhang LM, Wang H (2013). Evaluation of the immunosuppressive activity of artesunate in vitro and in vivo. Int Immunopharmacol.

[34] Jiang W, Cen Y, Song Y, Li P, Qin R, Liu C (2016). Artesunate attenuated progression of atherosclerosis lesion formation alone or combined with rosuvastatin through inhibition of pro-inflammatory cytokines and pro-inflammatory chemokines. Phytomedicine.

[35] Mo HY, Wang LF, Zhang LH (2012). Effects of artesunate on tumor necrosis factor alpha and chemotactic factors in the serum and the synoviocyte culture supernate of collagen-induced arthritis rats. Zhongguo Zhong Xi Yi Jie He Za Zhi.

[36] Anthony SJ, Gilardi K, Menachery VD, Goldstein T, Ssebide B, Mbabazi R (2017). Further evidence for bats as the evolutionary source of Middle East respiratory syndrome coronavirus. MBio.

[37] Hu B, Zeng LP, Yang XL, Ge XY, Zhang W, Li B (2017). Discovery of a rich gene pool of bat SARS-related coronaviruses provides new insights into the origin of SARS coronavirus. PLoS Pathog.

[38] Canton J, Fehr AR, Fernandez-Delgado R, Gutierrez-Alvarez FJ, Sanchez-Aparicio MT, Garcia-Sastre A (2018). MERS-CoV 4b protein interferes with the NF-kappaB-dependent innate immune response during infection. PLoS Pathog.

[39] Li Q, Verma IM (2002). NF-kappaB regulation in the immune system. Nat Rev Immunol.

